# KIF5A and the contribution of susceptibility genotypes as a predictive biomarker for multiple sclerosis

**DOI:** 10.1007/s00415-020-10373-w

**Published:** 2021-01-23

**Authors:** Kelly Hares, K. Kemp, S. Loveless, C. M. Rice, N. Scolding, E. Tallantyre, N. Robertson, A. Wilkins

**Affiliations:** 1 MS and Stem Cell Group, Institute of Clinical Neurosciences, Bristol Medical School: Translational Health Sciences, University of Bristol, Clinical Neurosciences Office, 1st Floor, Learning and Research Building, Southmead Hospital, Bristol, BS10 5NB UK; 2 Division of Psychological Medicine and Clinical Neuroscience, School of Medicine, Cardiff University, Cardiff, UK

**Keywords:** Axonal loss, Biomarkers, Cerebrospinal fluid, KIF5A, Multiple sclerosis, Single nucleotide polymorphism

## Abstract

There is increasing interest in the development of multiple sclerosis (MS) biomarkers that reflect central nervous system tissue injury to determine prognosis. We aimed to assess the prognostic value of kinesin superfamily motor protein KIF5A in MS by measuring levels of KIF5A in cerebrospinal fluid (CSF) combined with analysis of single nucleotide polymorphisms (SNPs; *rs12368653* and *rs703842*) located within a MS susceptibility gene locus at chromosome 12q13–14 region. Enzyme-linked immunosorbent assay was used to measure KIF5A in CSF obtained from two independent biobanks comprising non-inflammatory neurological disease controls (NINDC), clinically isolated syndrome (CIS) and MS cases. CSF KIF5A expression was significantly elevated in progressive MS cases compared with NINDCs, CIS and relapsing–remitting MS (RRMS). In addition, levels of KIF5A positively correlated with change in MS disease severity scores (EDSS, MSSS and ARMSSS), in RRMS patients who had documented disease progression at 2-year clinical follow-up. Copies of adenine risk alleles (AG/AA; *rs1236865*3 and *rs703842*) corresponded with a higher proportion of individuals in relapse at the time of lumbar puncture (LP), higher use of disease-modifying therapies post LP and shorter MS duration. Our study suggests that CSF KIF5A has potential as a predictive biomarker in MS and further studies into the potential prognostic value of analysing MS susceptibility SNPs should be considered.

**Electronic supplementary material:**

The online version of this article (10.1007/s00415-020-10373-w) contains supplementary material, which is available to authorized users.

## Introduction

Multiple sclerosis (MS) is an immune-mediated inflammatory and neurodegenerative disease, presenting with clinical relapses [associated with inflammatory lesions within the central nervous system (CNS)], and/or progressive neurological decline (associated with axonal loss and neurodegeneration) [1]. Due to the complex, multifaceted pathophysiology of MS, predicting prognosis at disease onset, which impacts on the choice of disease-modifying therapy (DMT), remains challenging and imprecise. Current measures of disease activity include MRI analysis of lesion load and relapse rates, which reflect inflammatory aspects of the disease but do not correlate well with progressive disability [2]. Consequently, there is increasing interest in the development of MS fluid biomarkers that reflect CNS tissue injury to determine prognosis and/or monitor efficacy of treatment. Both radiological and post-mortem human tissue studies have already revealed axonal injury and loss in MS are closely linked to disability accumulation [1, 3, 4], thus axonal proteins may represent a potential avenue for reliable biomarker development.

Neurofilaments (NF) are major structural proteins of the axonal cytoskeleton, whose phosphorylation improves structural stability [5]. NFs in the cerebrospinal fluid (CSF) are considered to be a reliable marker of neuroaxonal damage [6] and many studies have highlighted NF-light (NF-L) as a prognostic and treatment-responsive biomarker in MS [7, 8]. However, multi-centre validation of these findings and determining age-dependent cut-off values for NF-L expression are required before consideration in clinical practice [9].

Both hypo- and hyper- phosphorylation of NFs within axons are recognised as pathological hallmarks of MS [10, 11] and it is believed dysregulated axonal transport could be a catalyst for aberrant protein phosphorylation and accumulation [12, 13]. The majority of anterograde axonal transport is governed by kinesin superfamily motor proteins (KIFs) [14]. KIF5A is the main kinesin subtype involved in anterograde transport of phosphorylated NFs [12, 15].

In humans, a missense mutation within the KIF5A gene (N256S) causes an autosomal dominant form of hereditary spastic paraplegia, known as SPG10. This disease is characterised pathologically by axonal loss in the corticospinal tract [16]. Another missense mutation in the KIF5A gene has been linked to Charcot-Marie-Tooth disease (CMT-2A); an inherited peripheral axonopathy [17]. More recently, hot-spot mutations in KIF5A have also been shown to cause familial amyotrophic lateral sclerosis (ALS) [18]. In addition to point mutations, several genome-wide association studies (GWAS) have identified single nucleotide polymorphisms (SNPs; *rs12368653*, *rs703842, rs10431552, rs10877013* and *rs6581155*) at chromosome 12q13–14. This region spans 17 candidate genes, including KIF5A that confer susceptibility to MS [19–21]. Our previous studies have found reduced KIF5A expression in post-mortem MS brain; levels of which appear to be influenced by MS susceptibility SNPs (*rs12368653* and *rs703842*) [22, 23]. In addition, we have shown that these MS susceptibility SNPs are linked to increased markers of axonal injury in MS tissue [23]. In this study, we aimed to determine the prognostic value of measuring CSF KIF5A levels in conjunction with the presence of MS susceptibility SNPs (*rs12368653* and *rs703842)* in people with MS.

## Methods

### Patient cohort and CSF sampling

CSF samples were obtained from two independent biobanks (Bristol and Cardiff). The Bristol cohort was used for analysis of KIF5A protein in CSF from people with MS and idiopathic intracranial hypertension (IIH). Ethical approval was received from the South West—Frenchay Research Ethics Committee (REC: 09/H0107/72) for lumbar puncture (LP) collection of CSF from patients with suspected CNS inflammation from neurology clinics based at Southmead Hospital, Bristol, with written consent. Patients with a diagnosis of relapsing–remitting MS (RRMS; *n* = 38), primary progressive MS (PPMS; *n* = 6) or secondary progressive MS (SPMS; *n* = 3), at the time of LP, were included in the study, as classified by revised 2017 McDonald criteria [24]. CSF was removed from patients with IIH for therapeutic relief and collected as a waste product, as per local REC recommendations. No clinical data was collected on IIH patients. These samples served as a non-inflammatory neurological disease control (NINDC *n* = 49). CSF was processed within 2hrs of collection, centrifuged at 3000×*g* for 10 min at room temperature and the supernatant separated into 300 µL aliquots, before storage at − 80 °C.

The Cardiff cohort had comprehensive clinical data available for samples and paired DNA samples for analysis of patient genotype. This cohort was used to analyse KIF5A in CIS and MS, in relation to a range of independent cohort variables including genotype. CSF and DNA samples were requested and received from the Welsh Neuroscience Research Tissue Bank (University Hospital of Wales, Cardiff, UK; REC: 14/WA/0073). Patients with a diagnosis of clinically isolated syndrome (CIS; *n* = 27), RRMS (*n* = 67), PPMS (*n* = 6) or SPMS (*n* = 5), according to contemporary diagnostic criteria at the time of LP for suspected neuroinflammation, were included in the study. No patients were receiving DMT or steroid therapy before LP. CSF was processed within 1 h of collection, centrifuged at 4400×*g* for 10 min at 4 °C and the supernatant separated into 300 µL aliquots, before storage at − 80 °C.

### Power calculation for SNP genotype and patient genotyping

A power calculation to determine required samples sizes was performed prior to this study. The power analysis was based on clinically significant differences detected in KIF5A protein expression between MS patients grouped by *rs703842* SNP alleles (GG vs AA) [23]. In this study the calculated standardised difference in KIF5A expression was 0.87. Using two-sample inference of means, case numbers required to detect similar change in KIF5A protein expression at 0.80 power and 0.05 significance was 22 patients per group [25].

Within the Cardiff cohort, most participants who had donated CSF had already been genotyped for *rs703842* and *rs12368653*. For both SNPs, adenine (A) represents the MS risk allele, with the alternative allele being guanine (G). gDNA was provided for 18 cases without genotype data and an additional 6 positive control genotype samples for *rs703842* (AA, AG and GG) and *rs12368653* (AA, AG and GG). 5 µL of diluted gDNA (2 ng/µL) was added in duplicate to a MicroAmp^®^ Fast Optical 96-Well Reaction Plate (ThermoFisher Scientific), including positive control samples and no template controls. 20 × TaqMan^®^ SNP Genotyping Assays (*rs12368653* or *rs4646536* (proxy for *rs703842;* linkage disequilibrium *r*^2^ = 1 [20]), ThermoFisher Scientific) were diluted in DNase-free H_2_O and 2 × qPCRBIO Genotyping Mix Hi-ROX (PCR Biosystems) and added to the appropriate samples wells to achieve a total volume of 20 µL. Genotyping was performed on a StepOnePlus™ Real-Time PCR system with clustering algorithm software (ThermoFisher Scientific), on a FAST ramp speed.

### Enzyme-linked immunosorbent assay (ELISA)

Levels of KIF5A protein in CSF were detected using commercially validated human ELISA kit for KIF5A (BT-Labs; E2780Hu; sensitivity 4.93 ng/L), as per manufacturers’ instructions. The kit had an intra- and inter-assay coefficient of 8% and 10% respectively (*n* = 3). CSF was defrosted shortly before the assays and repeated cycles of thawing and re-freezing avoided. Protein standards, samples and blanks were run in duplicate. 40 µL of undiluted CSF was loaded per well, respectively. Plates included the following positive and negative controls; MS white matter brain homogenate, blank (sample diluent and PBS) and biotin antibody negative. Protein absorbance was read at 450 nm using a FLUOstar OPTIMA plate reader 213 (BMG labtech, Aylesbury, BUCKS, UK). The associated OPTIMA software programme was used to interpolate sample protein concentrations from the respective standard curves generated by serially diluting kit protein standards.

### Statistics

Frequency distributions were examined using the *χ*^2^ contingency test. Data normality was tested using the Shapiro–Wilk test. Where clinical data was skewed, the median was used for data analysis. Assessment and appropriate removal of outliers from KIF5A ELISA data was performed using the mean ± 2 × standard deviation. Univariate protein analysis was performed using GraphPad Prism5™ (GraphPad Software Inc.; San Diego, USA). Parametric one-way ANOVA with post-hoc Bonferroni, or non-parametric Kruskal–Wallis with post-hoc Dunn’s test or Spearman’s rank correlation coefficient, as appropriate, was used to analyse KIF5A protein expression, in relation to disease phenotype and other independent cohort variables. For univariate tests, values of *p* < 0.05 were considered statistically significant. Multiple regression analysis was performed using STATA v12 (StataCorp LLC; Texas, USA). For multivariate analysis KIF5A protein levels was the dependent variable which was analysed against several independent cohort variables. Where necessary, data were transformed to normality before regression analysis. For multivariate analysis, an *α* 0.05 cut-off level was used to determine statistical significance and Bonferroni correction applied to allow for multiple testing and Type I errors.

## Results

### Cohort variances

Within the Bristol MS cohort, CSF sample storage duration ranged from 1 to 10 years (mean 5 years ± 2). MS cases ranged in age from 21 to 64 years (mean 41 years ± 12; *n* = 47) (Table 1). There was a significant difference in the average age of the RRMS cohort (mean 38 years ± 11) compared with the progressive MS cohort (PPMS and SPMS; mean 52 years ± 7) (*p* < 0.001; Supplementary Fig. 1). As a result of ethical constraints, no data was available on age or sex for IIH cases. Within the Cardiff cohort, sample storage ranged from 1 to 13 years (mean 6 years ± 4). Individuals ranged in age from 17 to 70 years (mean 41 years ± 12; *n* = 106). The MS subtype was unknown in one case, which excluded it from further analysis (Table 2). Like Bristol, there was a significant difference in the average age of patients with progressive MS (PPMS and SPMS; mean 55 years ± 9), compared with CIS (37 years ± 12) and RRMS (mean 40 years ± 10) (*p* < 0.001; Supplementary Fig. 2).

**Table 1:** Clinical characteristics of the Bristol cohort

Bristol cohort variables	*n*
Sample storage [mean ± SD (range)]	96 [5 years ± 2 (1–10)]
Sex (F/M)	31/16
IIH	49
RRMS [mean age ± SD (range)]	38 [38 years ± 11(21–64)]
PPMS [mean age ± SD (range)]	6 [52 years ± 5 (42–57)]
SPMS [mean age ± SD (range)]	3 [52 years ± 11 (40–61)]

*IIH* idiopathic intracranial hypertension, *PPMS* primary progressive multiple sclerosis, *RRMS* relapsing–remitting multiple sclerosis, *SPMS* secondary progressive multiple sclerosis

**Table 2:** Clinical characteristics of the Cardiff cohort

Cardiff cohort variables	*n*
Sample storage [mean ± SD (range)]	106 [6 years ± 4 (1–13)]
Sex (F/M) Ethnicity (WB/Cau)	(75/31) (58/18)
CIS [mean age ± SD (range)]	27 [37 years ± 12 (17–61)]
RRMS [mean age ± SD (range)]	67 [40 years ± 10 (26–70)]
PPMS [mean age ± SD (range)]	6 [54 years ± 10 (38–68)]
SPMS [mean age ± SD (range)]	5 [56 years ± 8 (45–64)]
*rs703842* (GG/AG/AA) CIS *rs703842* (GG/AG/AA) RRMS *rs703842* (GG/AG/AA) PPMS *rs703842* (GG/AG/AA) SPMS *rs703842* (GG/AG/AA)	(13/45/48) (3/11/13) (8/30/29) (1/4/1) (0/3/2)
*rs12368653* (GG/AG/AA) CIS *rs12368653* (GG/AG/AA) RRMS *rs12368653* (GG/AG/AA) PPMS *rs12368653* (GG/AG/AA) SPMS *rs12368653* (GG/AG/AA)	(33/55/18) (7/13/7) (22/34/11) (1/5/0) (2/3/0)
First inter-attack interval [median (range)]	66 [2 years (0.1–45.4)]
MS duration [median (range)]	84 [2 years (0.1–45.9)]
EDSS [median (range)]	87 [3.0 (0–6.5)]
DMT (yes/no)	40/48
Relapse (yes/no)	5/57
MSSS [median (range)] ARMSSS [median (range)]	74 [2.75 (0.01–9.59)] 87 [4.68 (0.29–9.05)]

*AA* homozygous adenine, *AG* heterozygous adenine/guanine, *ARMSSS* age-related multiple sclerosis severity scale, *Cau* Caucasian, *CIS* clinically isolated syndrome, *DMT* disease-modifying therapy, *EDSS* expanded disability status scale of Kurtze, *GG* homozygous guanine, *LP* lumbar puncture, *MSSS* multiple sclerosis severity scale, *PPMS* primary progressive multiple sclerosis, *RRMS* relapsing remitting multiple sclerosis, *SPMS* secondary progressive multiple sclerosis, *WB* white British

### CSF KIF5A is elevated in MS CSF compared with non-inflammatory neurological disease control

**Fig. 1:**
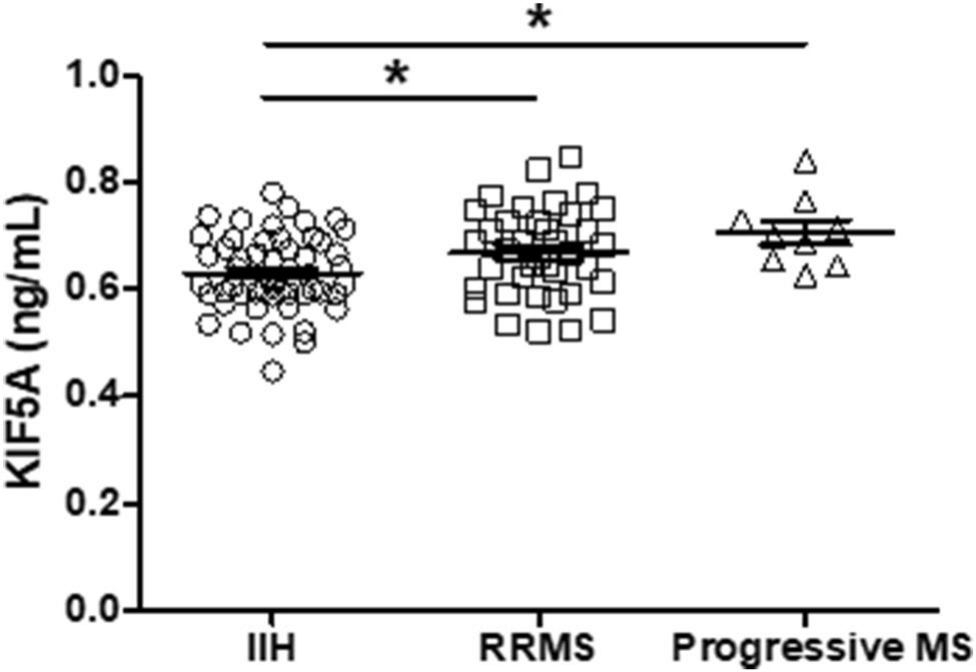
KIF5A levels are elevated in MS CSF compared with non-inflammatory neurological disease control (NINDC) in the Bristol cohort. Levels of KIF5A measured by ELISA are significantly increased in RRMS (*n* = 38) and progressive MS (*n* = 9) when compared to NINDC (IIH) (*n* = 48). Values represented as mean ± SEM. Statistical test used: one-way ANOVA with post-hoc Bonferroni; *p < 0.05. *IIH* idiopathic intracranial hypertension, *KIF* kinesin superfamily motor protein, *RRMS* relapsing–remitting multiple sclerosis

The Bristol cohort was used to determine differences in KIF5A CSF levels between NINDC (IIH) and MS. KIF5A levels were significantly higher in RRMS and progressive MS compared with IIH (*p* < 0.05; Fig. 1). Multivariate analysis with Bonferroni correction for type I errors, confirmed the finding with progressive MS (*p* < 0.01) but not RRMS *p* < 0.05; Table 3a). In addition, it showed no effect of IIH or MS sample storage duration on CSF KIF5A expression (*p* = 0.26; Table 3a). No data was available for age or sex of IIH but within the MS cohort, there was no effect of age (*p* = 0.46), sex (*p* = 0.48) or sample storage duration (*p* = 0.15) on MS CSF KIF5A expression (*n* = 47; Table 3b).

**Table 3:** Multivariate analysis of IIH sample storage and MS cohort variables on KIF5A expression in the Bristol cohort

a								
Protein	*n*	Variables	Coefficient	Standard error	*t*	*p* value	95% Confidence intervals	
KIF5A (ng/mL)	96	IIH vs RRMS	0.040	0.017	− 2.33	0.022	0.006	0.074
		IIH vs progressive MS	0.076	0.025	3.03	**0.003**	0.026	0.125
		RRMS vs progressive MS	0.036	0.027	1.34	0.184	− 0.017	0.088
		IIH and MS storage time	0.004	0.003	1.13	0.260	− 0.003	0.011

b								
Protein	*n*	Variables	Coefficient	Standard error	*t*	*p* value	95% confidence intervals	
KIF5A (ng/mL)	47	MS storage time	0.006	0.004	1.47	0.150	− 0.002	0.015
		MS age	− 0.001	0.001	− 0.74	0.462	− 0.003	0.001
		MS sex	− 0.021	0.029	− 0.72	0.477	− 0.078	0.037

Bonferroni correction to the required *αλπηα* 0.05 cut-off for significance has been applied. Significant associations highlighted in bold

*IIH* idiopathic intracranial hypertension, *KIF* kinesin superfamily motor protein, *MS* multiple sclerosis, *RRMS* relapsing–remitting multiple sclerosis

### CSF KIF5A expression is significantly elevated in progressive MS compared with CIS and RRMS

**Fig. 2:**
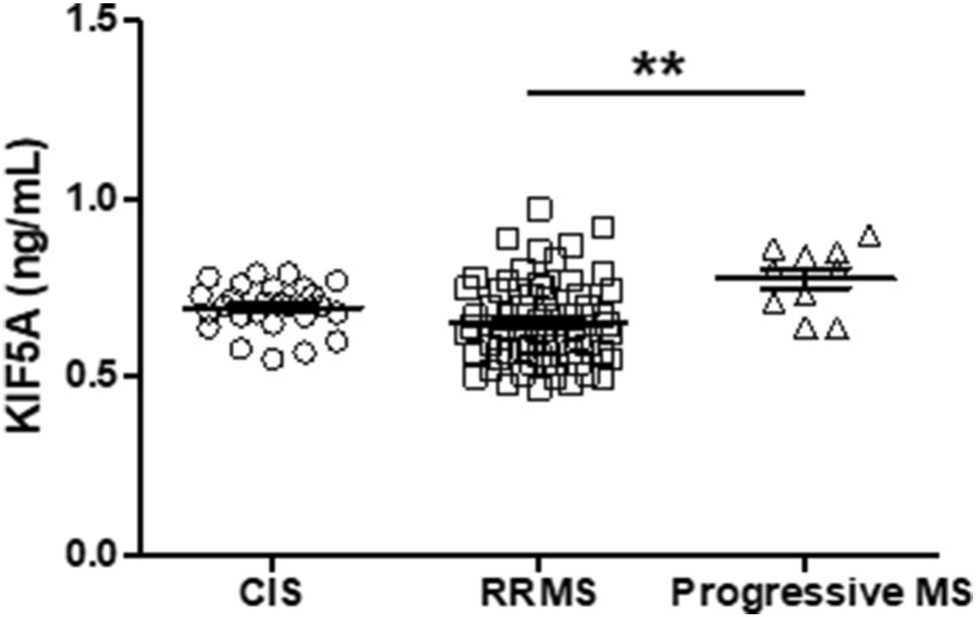
CSF KIF5A levels are elevated in progressive MS in the Cardiff cohort. KIF5A protein expression in cerebrospinal fluid samples were detected using ELISA. There was a significant increase in KIF5A expression in progressive MS cases (*n* = 10) compared with RRMS (*n* = 63) but not CIS (*n* = 26). Values represented as mean ± SEM. Statistical test used: one-way ANOVA with post-hoc Bonferroni; **p < 0.01. *CIS* clinically isolated syndrome, *KIF5A* kinesin superfamily motor protein 5A, *RRMS* relapsing–remitting multiple sclerosis

**Fig. 3:**
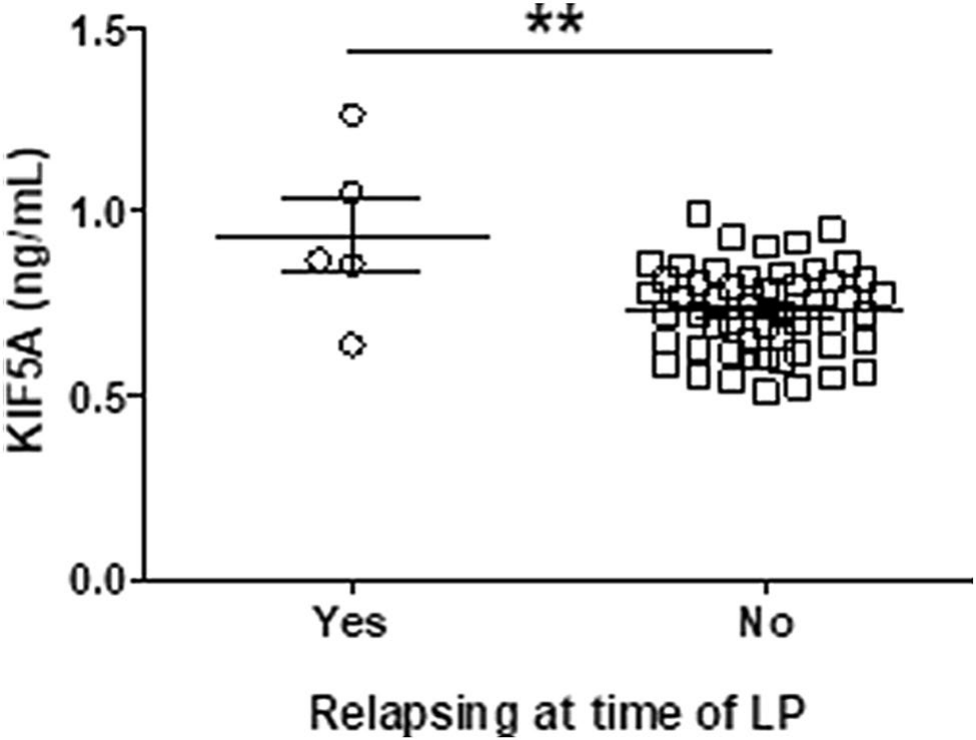
CSF KIF5A levels are elevated in MS relapse in the Cardiff cohort. KIF5A levels in MS CSF detected using ELISA are significantly higher in patients relapsing at the time of LP (*n* = 5) compared to those who are not (*n* = 54). Values expressed as mean ± SEM. Statistical test used: two-tailed T-test; **p < 0.01. *KIF5A* kinesin superfamily motor protein 5A, *LP* lumbar puncture

**Fig. 4:**
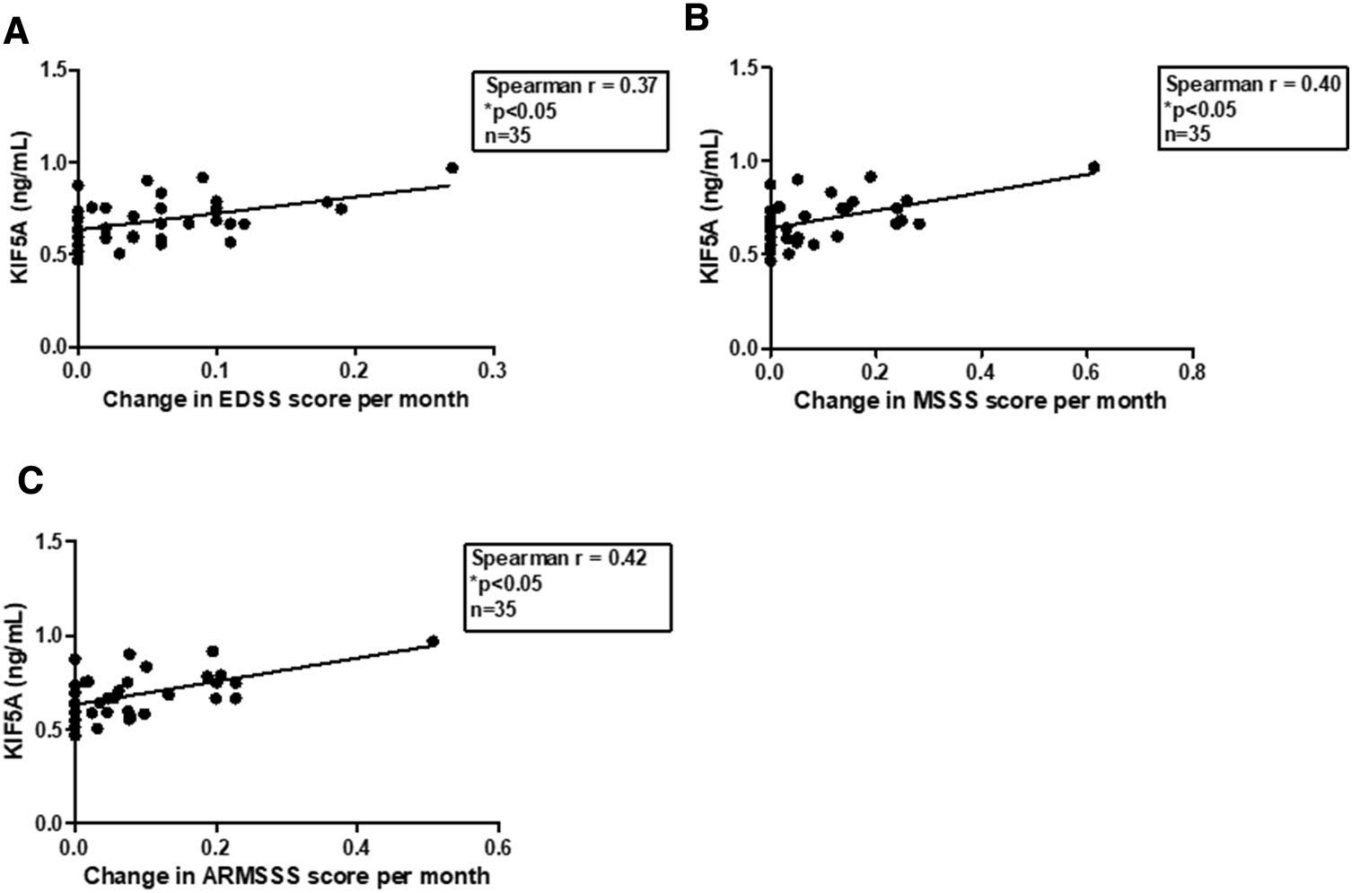
Levels of CSF KIF5A positively correlate with measures of disease severity in 2-year follow-up of relapsing–remitting patients within Cardiff cohort (*n* = 35). Levels of CSF KIF5A measured by ELISA significantly correlate with change in EDSS (**a**), MSSS (**b**) and ARMSSS (**c**) score as recorded from LP and average 24-month follow-up (range 13–40 months). KIF5A values expressed as mean. Statistical test used: non-parametric Spearman’s rank correlation coefficient, *p < 0.05. *ARMSSS* age-related multiple sclerosis severity score, *EDSS* expanded disability status score, *KIF5A* kinesin superfamily motor protein 5A, *MSSS* multiple sclerosis severity score

Detailed clinical documentation for Cardiff cohort CSF samples was used to analyse differences in KIF5A expression between CIS and MS subtypes. Four outliers within the KIF5A data were removed from cohort multivariate analysis. In univariate analysis, levels of KIF5A detected in CSF were significantly higher in patients with progressive MS compared with RRMS (*p* < 0.01; Fig. 2). This effect was confirmed in multivariate analysis (*p* < 0.01), which also revealed a significant difference in levels of CSF KIF5A between CIS and progressive MS samples when accounting for additional independent cohort variables (*p* < 0.01; Table 4). There was no effect of age (*p* = 0.94), sex (*p* = 0.74) or sample storage duration (*p* = 0.08) on KIF5A expression (Table 4). Initial analysis also showed levels of KIF5A in MS CSF were significantly higher in patients who were in relapse at time of LP (*p* < 0.01; Fig. 3) but the finding was not supported in the multivariate model (*p* = 0.27; Table 4). In multivariate analysis, no significant associations were found with MS duration, first inter-attack interval, multiple sclerosis severity scale (MSSS) and age-related MSSS (ARMSSS; Table 4). When studying KIF5A expression in relation to disease severity scores (EDSS, MSSS and ARMSS) over 2-year follow-up in the RRMS cohort there was no significant correlation (*n* = 67). However, when analysing RRMS cases that showed progression over 2-year follow-up there was a significant positive correlation with KIF5A levels (*n* = 35; Fig. 4). The mean and median follow-up assessment interval was 24 months (range 13–40 months; *n* = 35). Cases were excluded from analysis where initial EDSS assessment was more than 4 months post LP (*n* = 10).

**Table 4:** Multiple regression analysis of KIF5A within the Cardiff cohort

Protein	*n*	Variables	Coefficient	Standard error	*t*	*p* value	95% Confidence intervals	
KIF5A (ng/mL)	101	CIS vs RRMS	0.081	0.058	1.39	0.176	− 0.039	0.200
		CIS vs progressive MS	0.333	0.088	3.82	**0.001**	0.154	0.515
		RRMS vs progressive MS	0.254	0.077	3.29	**0.003**	0.095	0.413
	102	Age at LP	− 0.005	0.004	− 1.18	0.250	− 0.013	0.004
	102	Storage time	− 0.012	0.008	− 1.59	0.125	− 0.028	0.004
	102	Sex	0.026	0.058	0.45	0.654	− 0.094	0.147
	102	*rs703842* GG vs AA	0.173	0.157	1.10	0.281	− 0.151	0.497
		*rs703842* AG vs AA	0.151	0.060	2.53	**0.019**	0.028	0.275
	102	*rs12368653* GG vs AA	0.023	0.090	0.26	0.799	− 0.163	0.210
		*rs12368653* AG vs AA	0.063	0.072	0.87	0.391	− 0.085	0.211
	70	MSSS	0.015	0.017	0.91	0.371	− 0.020	0.051
	83	ARMSSS	− 0.030	0.022	− 1.35	0.191	− 0.765	0.016
	60	Relapsing at LP	− 0.139	0.124	− 1.12	0.273	− 0.394	0.116
	67	First inter-attack interval	0.000	0.004	0.12	0.904	− 0.007	0.008

Bonferroni correction to the required *αλπηα* 0.05 cut-off for significance has been applied. Significant associations highlighted in bold

*AA* homozygous adenine, *AG* heterozygous adenine/guanine, *CIS* clinically isolated syndrome, *ARMSSS* age-related multiple sclerosis severity score, *GG* homozygous guanine, *EDSS* expanded disability status score, *KIF* kinesin superfamily motor protein, *LP* lumbar puncture, *MSSS* multiple sclerosis severity score, *RRMS* relapsing–remitting multiple sclerosis

### MS risk SNPs are associated with MS disease activity

**Fig. 5:**
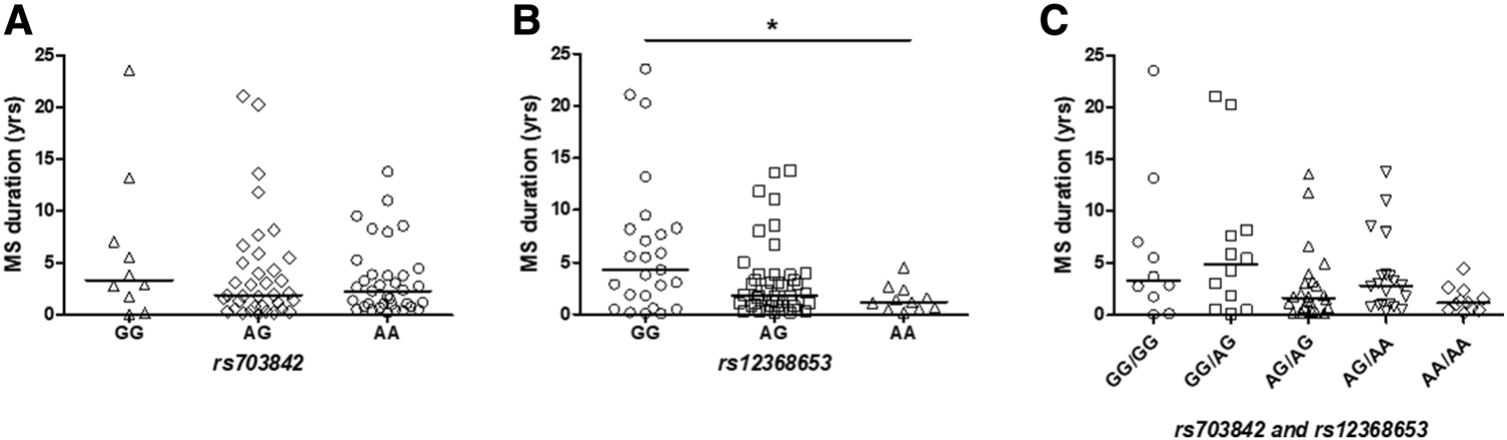
Influence of MS susceptibility genotypes on MS duration in the Cardiff cohort. No significant difference in MS duration in patients with MS susceptibility SNP *rs703842* (AG *n* = 36 and AA *n* = 35), compared to those without (GG *n* = 10; **a**). No significant difference in MS duration in patients heterozygous for MS susceptibility allele *rs12368653* (AG = 44) but significantly shorter duration in homozygous patients (AA *n* = 11), compared to those without (GG *n* = 25; **b**). No significant difference in MS duration in patients with multiple copies of susceptibility SNPs *rs703842* and *rs12368653* (AG/AG *n* = 24; AG/AA *n* = 20; AA/AA *n* = 11), compared to those without (GG/GG *n* = 10; GG/AG *n* = 12; **c**). Results expressed as median. Statistical test used: Kruskal–Wallis with post-hoc Dunn’s multiple comparison, *p < 0.05. *AA* homozygous adenine, *A/G* heterozygous adenine/guanine, *GG* homozygous guanine, *MS* multiple sclerosis

**Fig. 6:**
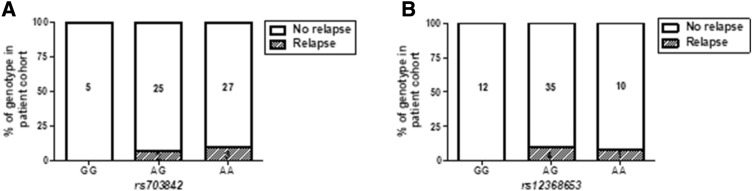
MS susceptibility genotypes in relation to cohort relapse incidence in the Cardiff cohort (*n* = 62). Patients with copies of susceptibility SNPs (AG/AA) *rs703842* (*χ*^2^ = 9.85, *p* < 0.01; **a**) and *rs12368653* (*χ*^2^ = 9.93, *p* < 0.01; B) have documented relapses at time of lumbar puncture compared with patients who have no copies of the susceptibility alleles (GG). Statistical test used: Chi-square test, **p < 0.01. *AA* homozygous adenine, *AG* heterozygous adenine/guanine, *GG* homozygous guanine

**Fig. 7:**
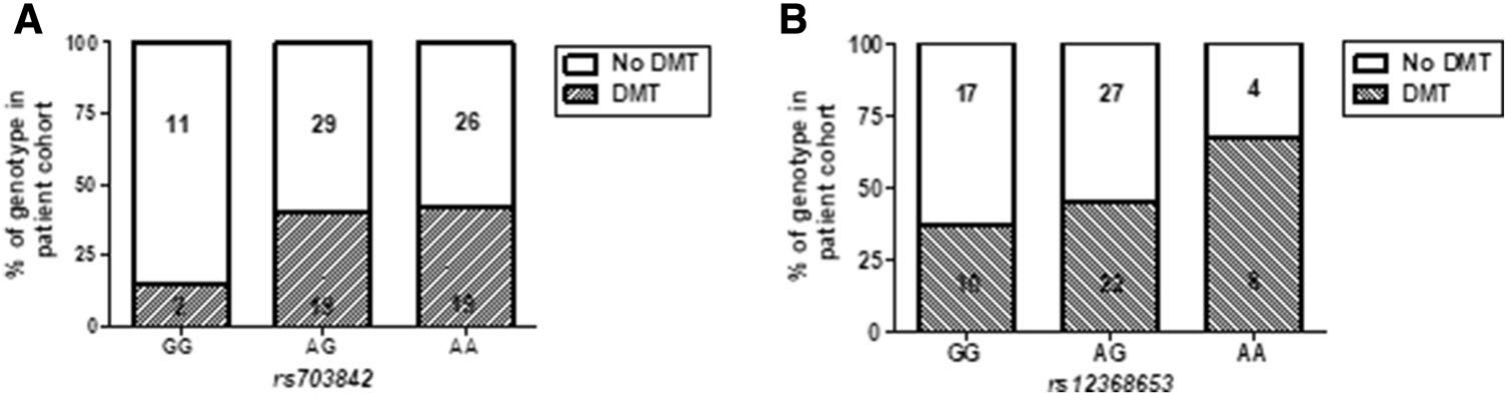
DMT incidence within MS susceptibility genotype subgroups in the Cardiff cohort (*n* = 88). A higher percentage of patients heterozygous (AG) or homozygous (AA) for MS risk SNPs *rs703842* (*χ*^2^ = 20.69, *p* < 0.001; **a**) and *rs12368653* (*χ*^2^ = 19.31, *p* < 0.001; **b**) received DMT compared to those without (GG). Statistical test used: Chi-square test, ***p < 0.001. *AA* homozygous adenine, *AG* heterozygous adenine/guanine, *DMT* disease-modifying therapy, *GG* homozygous guanine

Within the Cardiff cohort, we investigated associations between CSF KIF5A and SNPs (*rs703842* and *rs12368653*; both risk allele A) at chromosome 12q13–14 that are linked to MS susceptibility. Multivariate analysis showed a significant effect of patient *rs703842* genotype (AA vs AG) on CSF KIF5A levels, alongside disease subtype and a range of independent cohort variables (*p* < 0.05; Table 4). There was no significant difference in patient age between SNP susceptibility alleles [*rs703842* (*p* = 0.20) and *rs12368653 *(*p* = 0.07); *n* = 106, Supplementary Fig. 3]. There was no significant difference in MS duration (measured from symptom onset) in patients homozygous for *rs703842* risk SNP AA vs GG (*p* = 0.09; Fig. 5a) but a significantly shorter MS duration in patients homozygous for *rs12368653* risk SNP AA vs GG (*p* < 0.05; Fig. 5b). Combining both genotypes showed no significant difference in MS duration in patients homozygous for both SNPs (AA/AA) compared to those with no copies (GG/GG; *p* = 0.06; Fig. 5c). There was no significant difference in first inter-attack interval in patients homozygous for MS risk SNPs *rs703842* [*p* = 0.99, *n* = 64) and *rs12368653 *(*p* = 0.55, *n* = 65); Supplementary Fig. 4]. However, when each allele subset (GG, AG, AA) was normalised to correct for allele population frequency, only patients with copies of risk alleles (AG and AA) were documented as being in active relapse at the time of LP (*rs703842* *χ*^2^ = 9.85 and *rs12368653* *χ*^2^ = 9.93; *p* < 0.01, *n* = 62; Fig. 6). In addition, a significantly higher percentage of patients in the cohort with copies of risk alleles (AG and AA*)* received a DMT post LP, compared to those without copies (GG) (*rs703842* *χ*^2^ = 20.69 and *rs12368653* *χ*^2^ = 19.31; *p* < 0.001, *n* = 88; Fig. 7).

## Discussion

Novel prognostic biomarkers for MS are lacking. Proteins linked to inflammation such as myelin basic protein, have been analysed in CSF as a measure of disease activity but lack disease specificity. Axonal proteins are currently being studied as a more accurate reflection of neuronal damage and disease progression [26].

In this study, we have found significantly higher levels of KIF5A expression in CSF from progressive MS cases, validated in two independent CSF biobanks. In samples from the Bristol cohort, KIF5A was significantly elevated in progressive MS CSF compared with NINDC (IIH). One limitation of the Bristol cohort is the absence of clinical data for IIH, which restricts the multivariate analysis. However, it has the advantage of being a single NINDC diagnosis group, whereas other MS biomarker studies have heterogenous control populations [27, 28].

KIF5A is a neuronally enriched motor protein linked to anterograde axonal transport of various intracellular cargoes including phosphorylated NFs [12, 15]. Mouse models have shown KIF5A knockout is neonatal lethal. Post-natal targeting of the gene resulted in age-dependent sensory neurodegeneration and hind limb paralysis. This is attributed to NF accumulation in the cell body and axonal loss [12]. NF-L has been studied as a potential prognostic biomarker in MS and levels correlate well with markers of neuroinflammation including acute relapse and lesion load [27, 29]. However, there is confounding evidence on the utility of NF-L as a predictor of MS disability accumulation [30, 31].

In the Cardiff cohort, KIF5A was significantly elevated in CSF from progressive MS compared with RRMS and CIS in multivariate analysis. CSF KIF5A levels were significantly higher in patients who were in relapse at time of LP compared to patients who were not. This was not replicated in multivariate analysis, which may be due to the number of cases in relapse being underpowered. It is likely that acute inflammation during relapse could cause a higher influx of KIF5A into the CSF due to disruption at the blood brain barrier. This has also been found in studies examining NF-L as a marker of neuronal damage in MS [8].

Our finding of higher KIF5A levels in progressive MS patients compared with RRMS suggest CSF KIF5A levels could be predictive for underlying axonal loss and progressive disability. This is further supported by our data showing levels of KIF5A positively correlate with change in MS disease severity scores (EDSS, MSSS and ARMSSS), in RRMS patients who have documented disease progression at 2-year clinical follow-up. In progressive MS, patients become increasingly disabled over time as a result of axonal loss [3]. It is believed early axonal transport deficits that cause processes such as reduced transport of mitochondria and decreased ATP availability could initiate axonal damage and loss in MS [13]. One explanation for transport abnormalities could be reduced availability of KIFs. We have previously shown reduced KIF5A protein expression in MS white matter that inversely correlated with levels of APP and NFs [23], which are commonly found in axonal spheroids [10, 32]. Inflammatory mediators present in MS pathogenesis could also disrupt axonal transport; we have previously shown that nitric-oxide exposure reduces KIF5A expression in cultured neurons [33].

Other studies have highlighted that mutations in the KIF5A gene are directly linked to diseases with disturbed axonal transport and axonal loss [16–18]. GWAS have shown SNPs at chromosome 12q13–14 (which spans the KIF5A gene region), are linked to MS susceptibility [19–21]. Our previous human tissue studies have found significantly lowers levels of KIF5A in MS patients with copies of susceptibility SNPs (*rs12368653* and *rs703842*) that correlated with higher levels of dephosphorylated NFs, which are a hallmark of MS pathology [22, 23].

Results from our current study demonstrate CSF KIF5A levels are significantly different between patients heterozygous (AG) or homozygous (AA) for the *rs703842* MS susceptibility allele. Data available from the 1000Genomes project has estimated the minor allele (G) frequency within a European population (sample size 1006) at 0.320. This may explain why significant differences were not detected in patients without the SNP (GG) compared with homozygotes (AA). The estimated minor allele (A) frequency for *rs12368653* is 0.492. Patients homozygous for the *rs12368653* adenine MS susceptibility allele had significantly shorter MS duration (time from documented symptom onset to LP), compared with patients with no copies. This may indicate that patients have a more aggressive disease course, reaching diagnostic LP sooner. This theory is supported by additional findings from the Cardiff cohort which demonstrate that when genotype subsets (GG, AG, AA) are normalised to correct for allele population frequency, a higher percentage of patients with risk alleles (AG/AA) were in relapse at LP and went on to receive a DMT. Although of interest, it is important to note that the number of cases for *rs703842* GG (*n* = 13) and *rs12368653* AA (*n* = 18) were potentially underpowered (*n* < 22); genotype findings would therefore need validation in larger cohort studies to compensate for the minor allele frequencies of both *rs703842* and *rs12368653* [34].

Both *rs12368653* and *rs703842* SNPs are upstream of the KIF5A gene and form part of a locus control region, comprising 17 candidate genes [20, 21]. As a result of linkage disequilibrium, the non‐coding regions within several close genes can affect disease susceptibility and SNPs in non-protein coding regions can still affect gene splicing, transcription factor binding and mRNA degradation [20]. It is likely that the associations found with *rs12368653* and *rs703842* are not solely attributed to these individual SNPs and due to linkage disequilibrium, are linked with other SNPs around this gene locus at chromosome 12. Recent gene studies have indicated *rs70100*6 as a lead MS susceptibility SNP within region 29 of chromosome 12 [35, 36]. In future studies it would be important to analyse this SNP and other recent candidate SNPs within the same region to assess whether individual or clusters of SNPs are linked with disease outcomes. This could also be expanded to incorporate an in silico analysis using RNA expression data from brain, available in public databases.

Overall, this study has shown quantifiable differences in CSF KIF5A levels between NINDC, CIS, RRMS and progressive MS, suggesting levels are predictive of MS. In addition, CSF KIF5A levels correlated with measures of MS disease severity in RRMS patients who were documented as having increased disability scores over 2-year follow-up, suggesting KIF5A may have future prognostic potential in predicting disease progression. Of interest, differences in CSF KIF5A levels were detected based on SNP *rs703842,* which is located at chromosome 12q13–14; a gene locus linked to MS susceptibility. SNP genotypes *(rs703842* and *rs12368653*) alone showed differences with measures of MS disease activity, such as MS duration, proportion in relapse at the time of LP and DMT post LP. However, the findings require verification in a larger cohort and a full exploration of the prognostic value of all GWAS-identified MS susceptibility SNPs within this gene locus.

## Electronic supplementary material

Below is the link to the electronic supplementary material.

Supplementary material 1 (DOCX 53 kb)

## Data Availability

All data supporting the findings of this study are available within the article and at Bristol University data repository (data.bris).
