# Supplementary material for: KIF5A and the contribution of susceptibility genotypes as a predictive biomarker for multiple sclerosis
Source: J Neurol. 2021 Jan 23;268(6):2175–84. doi: 10.1007/s00415-020-10373-w (PMC8179895; doi:10.1007/s00415-020-10373-w)
Supplement: Supplementary file 1 — Supplementary material 1 (DOCX 53 kb) [file 415_2020_10373_MOESM1_ESM.docx]

**KIF5A and the contribution of susceptibility genotypes as a predictive biomarker for multiple sclerosis**

*Journal of Neurology*

Hares K^1^, Kemp K^1^, Loveless S^2^, Rice CM^1^, Scolding N^1^, Tallantyre E^2^, Robertson N^2^, Wilkins, A^1^

^1^ MS & Stem Cell Group, Institute of Clinical Neurosciences, Bristol Medical School: Translational Health Sciences, University of Bristol, Bristol, UK

^2^ Division of Psychological Medicine & Clinical Neuroscience, School of Medicine, Cardiff University, Cardiff, UK

**Corresponding author:** Dr Kelly Hares

Email: [Kelly.hares@bristol.ac.uk](mailto:Kelly.hares@bristol.ac.uk)

**Supplementary Figures**

**Supplementary Fig.1 Age of Bristol MS cohort.** Significant difference in age of RRMS patients (n=44) compared with progressive patients (n=10). Results expressed as mean +/- SEM. Statistical test used: two-tailed T-test; ***p<0.001. Abbreviations: LP: lumbar puncture; RRMS: relapsing-remitting multiple sclerosis

**Supplementary Fig.2 Age of Cardiff MS cohort.** Significant difference in age of progressive MS patients compared with RRMS and CIS. Results expressed as mean +/- SEM. Statistical test used: one-way ANOVA with post hoc Bonferroni; ***p<0.001. Abbreviations: CIS: clinically isolated syndrome; LP: lumbar puncture; RRMS: relapsing-remitting multiple sclerosis

**Supplementary Fig.3 MS susceptibility genotypes in relation to patient age in Cardiff cohort.** No significant difference in patient age at disease onset based on expression of MS risk alleles (*rs703842*; GG n=12, AG n=45, AA n=42 and *rs12368653*; GG n=32, AG n=50, AA n=17) (A and B). Results expressed as mean +/- SEM. Statistical test used: One-way ANOVA with post hoc Bonferroni. Abbreviations: AA: homozygous adenine; A/G: heterozygous adenine/guanine; GG: homozygous guanine; MS: multiple sclerosis

**Supplementary Fig.4 MS susceptibility genotypes in relation to first inter-attack interval in Cardiff cohort.** No significant difference in first inter-attack interval in patients homozygous for MS susceptibility SNPs (*rs703842*; AA n=31 and *rs12368653*; AA n=9) compared to heterozygotes (*rs703842*; AG n=27 and *rs12368653* AG n=37) and those without (*rs703842*; GG n=8 and *rs12368653*; GG n=21) (A and B). Results expressed as median. Statistical test used: Kruskal-Wallis with post hoc Dunn’s. Abbreviations: AA: homozygous adenine; AG: heterozygous adenine/guanine; GG: homozygous guanine; MS: multiple sclerosis; SNP: single nucleotide polymorphism
